# A lightweight multi-dimension dynamic convolutional network for real-time semantic segmentation

**DOI:** 10.3389/fnbot.2022.1075520

**Published:** 2022-12-15

**Authors:** Chunyu Zhang, Fang Xu, Chengdong Wu, Chenglong Xu

**Affiliations:** ^1^Faculty of Robot Science and Engineering, Northeastern University, Shenyang, China; ^2^Shenyang Siasun Robot & Automation Company Ltd., Shenyang, China; ^3^College of Intelligent Systems Science and Engineering, Harbin Engineering University, Harbin, China

**Keywords:** semantic segmentation, lightweight network, dynamic convolution, encoder-decoder, multi-dimension convolution

## Abstract

Semantic segmentation can address the perceived needs of autonomous driving and micro-robots and is one of the challenging tasks in computer vision. From the application point of view, the difficulty faced by semantic segmentation is how to satisfy inference speed, network parameters, and segmentation accuracy at the same time. This paper proposes a lightweight multi-dimensional dynamic convolutional network (LMDCNet) for real-time semantic segmentation to address this problem. At the core of our architecture is Multidimensional Dynamic Convolution (MDy-Conv), which uses an attention mechanism and factorial convolution to remain efficient while maintaining remarkable accuracy. Specifically, LMDCNet belongs to an asymmetric network architecture. Therefore, we design an encoder module containing MDy-Conv convolution: MS-DAB. The success of this module is attributed to the use of MDy-Conv convolution, which increases the utilization of local and contextual information of features. Furthermore, we design a decoder module containing a feature pyramid and attention: SC-FP, which performs a multi-scale fusion of features accompanied by feature selection. On the Cityscapes and CamVid datasets, LMDCNet achieves accuracies of 73.8 mIoU and 69.6 mIoU at 71.2 FPS and 92.4 FPS, respectively, without pre-training or post-processing. Our designed LMDCNet is trained and inferred only on one 1080Ti GPU. Our experiments show that LMDCNet achieves a good balance between segmentation accuracy and network parameters with only 1.05 M.

## 1 Introduction

Semantic segmentation, widely used in the real world, classifies every pixel of a visual image. Semantic segmentation visualization uses different colors to distinguish different classes of objects effectively. Semantic segmentation is mainly used in scene analysis, including medical imaging, autonomous driving, and satellite maps. Semantic segmentation has become one of the most critical tasks in computer vision.

Fully convolutional networks (FCN) (Long et al., 2015) pioneered the end-to-end training of neural networks, and many semantic segmentation networks use a full convolution approach to network construction. U-Net (Ronneberger et al., 2015) adopts a symmetric network structure and fuses high-level and low-level semantic information in decoding. SegNet (Badrinarayanan et al., 2017) introduces a pooling operation with pixel indices to optimize segmentation details at the decoder stage. In order to achieve higher segmentation accuracy, high-precision networks such as DeepLab series (Chen et al., 2017a,b, 2018), APCNet (He et al., 2019), and CANet (Zhang et al., 2019) have been proposed one after another. In practical application scenarios, slow inference speed and many parameters are the main reasons semantic segmentation cannot be applied. On the Cityscape dataset (Cordts et al., 2016), networks that meet the 80% accuracy requirement have inference speeds below 10 FPS or model parameters over 100 M. Lightweight real-time semantic segmentation research is imminent.

**FIGURE 1:**
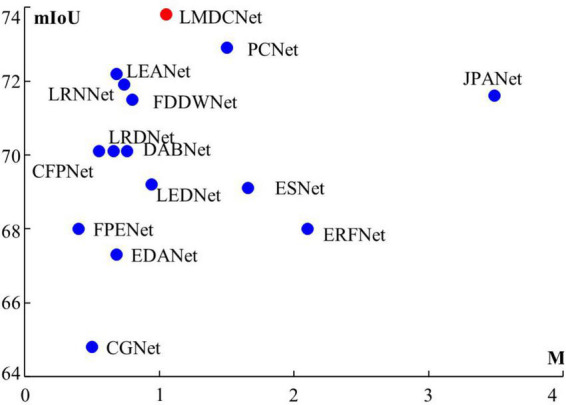
Accuracy of segmentation (mIoU) and network parameters (M) derived from Cityscapes test set. Clearly, our LMDCNet achieves the optimal balance between segmentation accuracy and parameters.

Lightweight real-time semantic segmentation requires a neural network that perfectly balances segmentation accuracy and parameter quantity. Typical lightweight real-time semantic segmentation networks are SegNet, ENet (Paszke et al., 2016), ICNet (Zhao et al., 2018), ERFNet (Romera Carmena et al., 2018), CGNet (Wu et al., 2020), BiSeNet (Yu et al., 2018), EDANet (Mehta et al., 2018), ESPNetV2 (Mehta et al., 2019), ESNet (Wang et al., 2019b), DABNet (Li G. et al., 2019), LEDNet (Wang et al., 2019a), DFANet (Li H. et al., 2019), FDDWNet (Liu et al., 2020), LRNNet (Jiang et al., 2020), LRDNet (Zhuang et al., 2021), JPANet (Hu et al., 2022), LEANet (Zhang et al., 2022) and our LMDCNet, As shown in Figure 1. When applying semantic segmentation, our first consideration is segmentation accuracy. PSPNet (Lv et al., 2021) pursues the fusion of multi-scale information, and SFNet (Lo et al., 2019) performs scale alignment of different features. The accuracy of these networks meets practical requirements, but the device’s computing power is too demanding. To overcome the memory requirement of the algorithm, ESPNetV2 proposes dilated convolutions for semantic segmentation, mainly to increase the receptive field. BiSeNetV2 adds a spatial branch to compensate for the loss of details in semantic segmentation. STDC-Seg designs the coding backbone network to reduce the number of parameters. These algorithms are less demanding on equipment but have poor segmentation accuracy.

**FIGURE 2:**
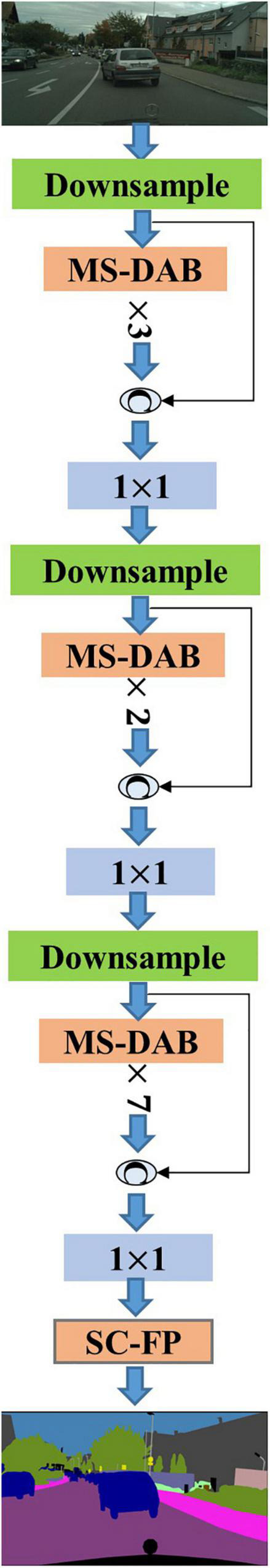
Overview architecture of the proposed LMDCNet.

**FIGURE 3:**
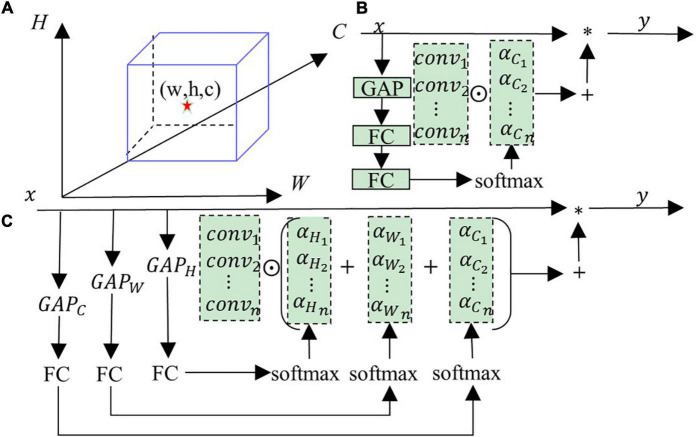
**(A)** Shows the feature map, **(B)** shows the typical dynamic convolution structure, **(C)** shows the multi-dimensional dynamic convolution structure (MDy-Conv).

This paper proposes a lightweight multi-dimensional dynamic convolutional network (LMDCNet) to solve the problem of unbalanced accuracy and parameters. The network adopts an asymmetric structure; the relevant details are shown in Figure 2. We design a new multi-dimensional dynamic convolution (MDy-Conv), which uses an attention mechanism for convolution and linearly combines multiple factorial convolutions to find a convolution kernel suitable for the current feature. Specifically, the operation flow is shown in Figure 3. We design the MS-DAB module to include MDy-Conv, residual connections, and channel shuffling operations. The encoder structure performs channel separation to reduce computational complexity. MDy-Conv is used to improve the coding performance, and channel shuffling improves the robustness of the network. Residual connections are used to reuse features and reduce the difficulty of training. The overall structure of the encoder is designed to achieve a perfect balance of encoding performance and parameters. We design a decoder with a feature pyramid structure, spatial attention, and channel attention: SC-FP. Feature Pyramid Module (FP) obtains multi-scale contextual information of features. Combining spatial and channel attention for efficient feature selection improves computational efficiency. To improve segmentation accuracy, SC-FP achieves a good balance between feature space details and computational network cost.

In brief, we have the following contributions:

1. A multi-dimensional dynamic convolution (MDy-Conv) is proposed. It adopts an attention mechanism for convolution and linearly combines multiple convolution kernels to find the best convolution kernel that conforms to the current feature encoding, thereby improving the encoding ability;
2. We propose a depth-asymmetric bottleneck module with multi-dimensional dynamic convolution and shuffling operations (MS-DAB module). It can effectively extract local and contextual information about features and fuse them. The MS-DAB module is far superior to similar modules in segmentation accuracy and parameters;
3. A feature pyramid (SC-FP module) with spatial and channel attention is proposed. The simplified feature pyramid incorporates multi-scale contextual information and uses spatial and channel attention for feature selection. Combining the two algorithms can extract more effective information during decoding and improve segmentation accuracy;
4. Using MS-DAB and SC-FP modules, create a Lightweight Multi-dimensional Dynamic Convolutional Network (LMDCNet). Evaluation results on the Cityscape dataset show that LMDCNet outperforms state-of-the-art networks, achieving the best balance between segmentation accuracy and parameters. On the CamVid dataset, the segmentation accuracy surpasses the current algorithms and reaches the top level.

## 2 Related work

In this section, we introduce algorithms related to lightweight real-time semantic segmentation, including the following: Dilated convolution, Attention mechanism, and Lightweight semantic segmentation network.

### 2.1 Dilated convolution

Dilated convolution is one of the standard methods for lightweight real-time semantic segmentation to reduce the number of parameters. This convolution has an additional hyper-parameter, called dilated rate, to represent the number of intervals in the kernel (e.g., the standard convolution is dilated rate 1). Yu and Koltun (2015) first applied dilated convolution to semantic segmentation algorithms. Later, the DeepLab series and DABNet, among others, borrowed the method further to improve the segmentation accuracy of semantic segmentation networks. Dilated convolution increases the convolutional receptive field and acquires contextual information. However, the dilated convolution produces grid effects due to adding 0 elements. Wang et al. (2018) proposed a hybrid dilated convolution that uses different dilated rates for each layer of the network so that the receptive field covers the entire region.

### 2.2 Attention mechanism

The role of the attention mechanism is to select features, highlight important information, and suppress unnecessary information. In order to make full use of limited visual information processing resources, attention is required to select features during information processing. SENet (Hu et al., 2018) (Squeeze and Excitation Network) is typical channel attention, and its purpose is to select feature channels. ECANet (Wang et al., 2020) is an enhanced version of SENet with a detailed explanation of channel attention. Convolutional block attention module (CBAM) (Woo et al., 2018) connects channel attention and spatial attention to form a hybrid attention mechanism.

### 2.3 Lightweight semantic segmentation network

Lightweight semantic segmentation network can accomplish on-device semantic segmentation tasks. Low computation, real-time reasoning, and accurate segmentation require lightweight semantic segmentation for practical tasks. At this stage, the devices that implement lightweight semantic segmentation are 1080Ti, 2080Ti, Titan, and 3080. Their processing power is 1080Ti < 2080Ti < Titan < 3080. We summarize three principles for designing lightweight semantic segmentation at this stage: (1) Improvement of the existing lightweight network backbone. For example, DFANet aims to use a lightweight classification network to encode semantic segmentation. Shuffle-Seg is an application of the lightweight classification network ShuffleNet in the direction of semantic segmentation. (2) Create a lightweight coding module as the coding base. For example, LEDNet uses only decomposed convolutional methods to design coding units. (3) Reduce the loss of segmentation details and increase the network coding branch. For example, BiSeNet designed a semantic segmentation network with spatial and context branches.

## 3 Materials and methods

In this section, we propose the LMDCNet network to balance the accuracy and the number of parameters for semantic segmentation. In Section “3.1 Multi-dimension dynamic convolution,” we propose multi-dimension dynamic convolution (MDy-Conv). We propose a depth-asymmetric bottleneck module with multi-dimension dynamic convolution and shuffling operations (MS-DAB module) and describe it in detail in Section “3.2 MS-DAB module.” In Section “3.3 SC-FP module,” we propose a feature pyramid module with spatial and channel attention (SC-FP module). Finally, we design the architecture of the whole network in Section “3.4 Network architecture”.

### 3.1 Multi-dimension dynamic convolution

Dynamic convolution has become the focus of attention in recent years. The output *y* of ordinary convolution is equal to the convolution operation performed by the convolution kernel *conv* and the input *x*, and * represents the convolution operation, as shown in Equation 1. Dynamic convolution is a convolution obtained by linearly combining multiple convolution kernels. The current feature obtains the weight in the combination process through correlation processing. As the input features change, the combined weight of the convolution also changes, so it is a dynamic convolution. CondConv (Yang et al., 2019) and DyConv (Chen et al., 2020) are typical dynamic convolutions whose structure is shown in Figure 3B. CondConv and DyConv use a modified SE (Squeeze-and-Excitation) attention structure to calculate convolution weights. The convolution kernel obtained by multiplying the weight with multiple convolutions and then adding them is dynamic convolution. The specific operation process is shown in Figure 3B. Then the output of a typical dynamic convolution follows Equation 2, where ⊙ represents the multiply add operation, and *a_C* represents the convolution combination weight vector obtained by processing in the channel-wise direction. *Conv* represents the list of convolution kernels. The combined weight of this dynamic convolution is derived from the channel direction of the feature, the information obtained is limited, the convolution kernel cannot be linearly combined, and the generated dynamic convolution could be more optimal.


(1) y=conv*x

(2) $$\text{y}=\left(\alpha_{C}⊙\text{Conv}\right)*\text{x}$$

(3) $$\text{y}=\left(\left\{\alpha_{W}+\alpha_{H}+\alpha_{C}\right\}⊙\text{Conv}\right)*\text{x}$$

As shown in Figure 3A, the feature map contains three dimensions, namely height (H), width (W), and channel (C). Locating a point in the feature map requires three dimensions to work together, and a single dimension cannot lock a point. Similarly, a single feature channel direction cannot determine the optimal convolution combination (i.e., weight), and three directions must work together. Based on the above arguments, we design a multi-dimensional dynamic convolution (MDy-Conv), and the detailed operation flow is shown in Figure 3C. The dynamic convolution we designed contains the information on the feature map’s three directions (H, W, and C), and the resulting convolution kernel combined weight is optimal. The specific description of the dynamic convolution generated by the feature *x* is as follows: (1) Perform global average pooling (GAP) on the three directions (height, width, and channel) of the feature *x* to obtain three tensors (*c* × 1 × 1, *h* × 1 × 1, *w* × 1 × 1), where (*c*, *w*, *h*) represent the channel, width, and height values, respectively; (2) They are sent to 3 fully connected layers (FC) and softmax, respectively, to obtain the exact size tensor (*r* × 1 × 1), where the size of *r* represents the number of convolutions participating in the calculation; (3) Add the three tensors to get the final convolution weight, and its size is also (*r* × 1 × 1); and (4) Multi-dimensional dynamic convolution (MDy-Conv) that performs multiplication and addition operations on *r* convolutions and convolution weights. The mathematical expression of multi-dimensional dynamic convolution is shown in Equation 3, (*a*_*C*_, *a*_*H*_, *a*_*W*_) represents the tensor obtained by feature *x* after global average pooling, fully connected layer, and softmax.

As shown in Figure 3, the differences between our multi-dimensional dynamic convolution and others are: First, we entirely use the information in the feature map to find the optimal solution for the combination of convolutions. In contrast, ordinary dynamic convolution only considers channel direction. Second, we use a single-layer fully connected layer, traditional dynamic convolution uses two layers, and we have fewer parameters. Third, the performance of our designed dynamic convolutional encoding is stronger than other dynamic convolutions, which we verified in comparative experiments.

### 3.2 MS-DAB module

**FIGURE 4:**
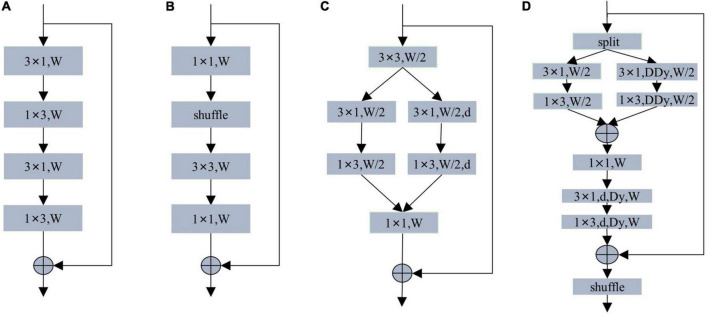
**(A)** Non-bottleneck-1D module. **(B)** ShuffleNet module. **(C)** DAB module. **(D)** Our MS-DAB module. W denotes the number of input channels. d denotes dilated convolution. DDy denotes depth-separable dynamic convolution. Dy denotes dilated dynamic convolution. For brevity, the batch normalization and activation functions are not marked.

The coding module of the lightweight real-time semantic segmentation network design pays more attention to the coding ability and the number of parameters. Most of the encoding modules adopt the structure of ResNet’s residual module. As shown in Figure 4, ERFNet designs a non-bottleneck-1D module using decomposed convolutions. ShuffleNet designs a lightweight real-time encoding model using group and depth-wise separable convolution. The DAB module uses asymmetric depth-wise separable convolution and asymmetric depth-wise dilated separable convolution.

Based on the above observations, our MS-DAB module design is shown in Figure 4D. First, we use a channel separation technique to segment the input features in the channel direction, thereby reducing the computational complexity. The depth-wise separable dilated convolution and dynamic convolution can improve the expressiveness of the model without increasing the network width and depth. Therefore, we replace the 3 × 3 convolutions in the first branch with 3 × 1 convolutions and 1 × 3 convolutions. We replace the standard 3 × 3 convolution in the second branch with 3 × 1 and 1 × 3 depth-wise multi-dimensional dynamic convolution. In order to achieve a better encoding effect, the feature maps of the two branches are spliced together, and 1 × 1 convolution is used to perform information fusion between feature map channels. In order to increase the receptive field of the module and obtain the contextual information of the feature, we add a 3 × 1 and a 1 × 3 multi-dimensional dilated dynamic convolution. Afterward, residual connections are used to improve feature utilization and simplify training. Finally, we use the shuffle operation in Figure 4B to enhance the robustness of the encoder.

Compared with the residual module of the same type, our MS-DAB module has the following advantages: First, we introduce MDy-Conv convolution in the residual module, which improves the encoding ability of the module; Second, the module adopts feature channel separation. The operation is separated from the convolution depth to reduce the computational complexity; Thirdly, the hollow multi-dimensional dynamic convolution is introduced to increase the receptive field of the encoder and improve the segmentation accuracy; Finally, channel shuffling and residual connection are used to improve the robustness of the network, reduce the difficulty of training.

### 3.3 SC-FP module

**FIGURE 5:**
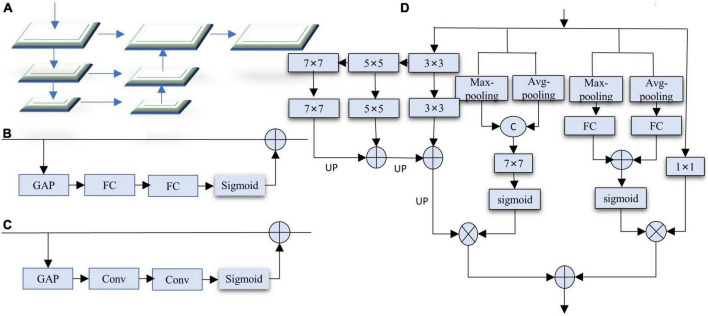
**(A)** Feature pyramid network (FPN). **(B)** Channel attention (CA). **(C)** Spatial attention (SA). **(D)** Our SC-FP module. For brevity, the batch normalization and activation functions are not marked.

The image segmentation scene is complex and changeable, and simple upsampling will lose details. Moreover, most lightweight real-time semantic segmentation adopts three coding stages, resulting in a too-small receptive field. Lightweight real-time semantic segmentation requires the decoding part to increase the receptive field, improve multi-scale information fusion, and reduce the loss of details. Therefore, we design a decoding module feature pyramid with spatial and channel attention (SC-FP module) that includes feature pyramid structure, spatial attention, and channel attention mechanisms. Feature pyramid structure can fuse multi-scale context information while increasing the receptive field of the network and reducing the loss of details. FPN proposes a feature pyramid structure, as shown in Figure 5A. FPN works well for multi-scale object recognition. However, too many layers exist in each encoding stage, resulting in an enormous computational burden. Channel Attention (CA) and Spatial Attention (SA) can perform feature selection on both channels and spaces, and the specific structures are shown in Figures 5B, C.

Based on the above observations, we designed the SC-FP module, as shown in Figure 5D. It integrates feature pyramid, channel attention, and spatial attention, effectively enhancing the ability to capture multi-scale contextual information and reducing the loss of image details. The decoder contains four branches: feature pyramid branch, channel attention branch, spatial attention branch, and channel compression branch. The feature pyramid branch comprises 3 × 3, 5 × 5, and 7 × 7 convolutions. Due to the smaller resolution of the features, using larger convolution kernels brings little computational burden. To further improve the performance, a channel attention branch is introduced. Channel attention consists of global max-pooling, global average-pooling, and two fully connected layers. Unlike other channel attention, we adopt a double pooling operation, which can obtain more channel information. The third branch is the feature channel compression branch, where the 1 × 1 convolution fuses the information between different channels to make the output channel equal to the segmentation category. Considering that the loss of details in lightweight real-time semantic segmentation seriously affects the segmentation accuracy, a spatial attention branch is introduced to integrate the global context. Spatial attention includes global average-pooling, global max-pooling, and 7 × 7 convolutions. Channel attention performs channel selection on the result of the 1 × 1 convolution, while spatial attention acts on the output of the pyramid to highlight detailed information. Finally, the two results are added point by point to generate the decoded feature map.

Our SC-FP module has the following advantages: First, it adopts a feature pyramid structure to increase the receptive field of the network, capture multi-scale context information, reduce the loss of details, and improve network performance. Second, it introduces a dual attention mechanism to integrate context information further, increase attention to detail information, and improve segmentation accuracy; Third, to reduce the computational burden, point-by-point multiplication or addition is used for feature fusion. Although a larger convolution kernel is used, the feature map resolution is lower and does not increase the computational complexity.

### 3.4 Network architecture

Our main objective in this work is to create a compact model that can strike the best balance between segmentation accuracy and network parameters. We propose the LMDCNet depicted in Figure 2 utilizing the SC-FP and MS-DAB modules to achieve this. The specific architecture of our LMDCNet, which has an asymmetric encoder-decoder, is displayed in Table 1.

**TABLE 1:** The detailed architecture of lightweight multi-dimensional dynamic convolutional network (LMDCNet).

Stage	Type	Channel	Output size
Encoder	Downsampling	32	512 × 256
	MS-DAB × 3	32	512 × 256
	Downsampling	64	256 × 128
	MS-DAB × 2	64	256 × 128
	Downsampling	128	128 × 64
	MS-DAB (*r* = 1)	128	128 × 64
	MS-DAB (*r* = 2)	128	128 × 64
	MS-DAB (*r* = 5)	128	128 × 64
	MS-DAB (*r* = 2)	128	128 × 64
	MS-DAB (*r* = 5)	128	128 × 64
	MS-DAB (*r* = 9)	128	128 × 64
	MS-DAB (*r* = 17)	128	128 × 64
Decoder	SC-FP	C	128 × 64
	Upsampling	C	1024 × 512

“Channel” denotes the number of output feature maps, and “C” is the number of classes. “Output size” denotes the output size with an input size of 1024 × 512.

In the encoder section of LMDCNet, we created three downsampling blocks and three encoder stages. The initial block in ENet, a cascaded output of 3 × 3 convolution with step 2 and a 2 × 2 pooling, serves as the downsampling block. The downsampling operation produces thumbnails of the corresponding images, enabling deeper networks to gather more contextual data while requiring less computational work. Downsampling, however, lowers spatial resolution, which typically results in a loss of spatial information and impacts the predictions’ outcomes. Therefore, to maintain a good balance, only three downsampling operations—for a total downsampling rate of eight—are carried out in our LMDCNet. The three, two, and seven MS-DAB modules comprise LMDCNet’s three encoder stages. We introduce dilated convolution in the MS-DAB module. To solve the grid problem, we follow the design concept of HDC (hybrid dilated convolution) when designing the dilation rates: First, the adjacent dilation rates cannot be greater than the common divisor of 1; Second, the dilation rates can be designed as a zigzag structure, such as (1, 2, 5, 2, 5, 7); Third, the final dilation rates should cover the maximum segmentation target. The specific design of the network is as follows: the dilation rates of the first stage and the second stage are set to 1, and the dilation rates of the third stage is set to (1, 2, 5, 2, 5, 9, 17).

Many lightweight real-time networks remove the decoder part, and proper decoding can improve network accuracy. The decoder includes the SC-FP module and the upsampling module; obviously, our network architecture is asymmetric. The SC-FP module contains feature pyramids and attention, which can refine the detailed information on segmentation and the selection of features. The feature map size does not match the input image size, and a bilinear interpolation algorithm is needed to recover the feature map resolution. The parameters of the decoder part are few but can effectively improve the segmentation accuracy. Our network has no complicated data processing links in the training process, and the number of parameters is only 1.05 M.

## 4 Experiments

In this section, we evaluate the performance of our designed LMDCNet on two challenging public datasets, the Cityscapes, and CamVid datasets. We first introduce the two datasets used in the experiments and the implementation details. The effectiveness of each LMDCNet component is then demonstrated using a series of ablation experiments on the Cityscapes validation set. Finally, we present evaluation results on the CamVid and Cityscapes test sets and comparisons with other lightweight real-time semantic segmentation networks.

### 4.1 Datasets

#### 4.1.1 Cityscape dataset

Cityscape dataset is a large dataset for semantic segmentation for training. The dataset contains 5000 finely labeled images and 20,000 coarsely labeled images. Usually, fine-labeled images are used for network training, and coarse images are used for network migration for pre-training. The resolution of the images is 1024 × 2048, and the default classification label is 19 classes. We compressed the image resolution to 512 × 1024 to improve the inference speed.

#### 4.1.2 CamVid dataset

CamVid dataset uses street scenes from video sequences as semantic segmentation training data. The dataset has 701 high-quality training images, of which 367 are the training set, 101 are the validation set, and 233 are the test set. The dataset contains 32 semantic categories, and the categories commonly used for network training are 11 categories. The resolution of the images is 720 × 960, and 360 × 480 is used in our training process.

### 4.2 Implementation details

#### 4.2.1 Environment configuration

The model creation and training were based on the Pytorch platform with CUDA 9.0 and cuDNN 7, and all experiments were conducted on a machine outfitted with an Intel i7-10700K CPU and a single NVIDIA GTX 1080Ti GPU (11G).

#### 4.2.2 Network training configuration

We did not employ any additional datasets as network preprocessing. We used small batch stochastic gradient descent (SGD) during the training process as the optimization function with a weight decay of 2e-4 and a momentum of 0.9. The batch processing size is 8 for the Cityscapes dataset and 16 for the CamVid dataset. The cross-entropy loss function is used for the loss function. The initial learning rate for the Cityscapes dataset is 4.5e-2, and the CamVid dataset is 1e-3 using the “poly” learning rate technique. The current epoch learning rate is *lr* = *init*_*lr* × (1 − *epoch*/max_*epoch*)^*power*^, where power is 0.9.

#### 4.2.3 Data augmentation

Data augmentation reduces the risk of training overfitting. Our experiments used the following methods for data augmentation: average subtraction, random level flipping, and random scaling. The scales of random scaling during training are 0.75, 1.0, 1.25, 1.5, 1.75, and 2.0.

#### 4.2.4 Evaluating indicator

The evaluation metrics of semantic segmentation include three aspects: segmentation accuracy, inference speed, and model size. Segmentation accuracy is measured by mean Intersection over Union (mIoU); inference speed is measured by the number of frames per second (FPS) processed in the image, and model size is measured by the number of statistically learnable parameters (M).

#### 4.2.5 Network performance balance indicator

We designed an optimal balance index to evaluate the accuracy and parameter amount of lightweight real-time semantic segmentation, and it is named as increment rate (IR). The most critical indicators of lightweight real-time semantic segmentation are segmentation accuracy, parameter amount and inference speed. Because the inference speed is related to the verification platform, the speed of comparing lightweight real-time semantic segmentation must be on a unified platform. The standard for evaluating the quality of a lightweight real-time semantic segmentation network is that the higher the accuracy, the lower the parameters, and the better the network. It is equivalent to an inverse relationship between the segmentation accuracy and the number of parameters. We must divide the accuracy by the number of parameters. Let us take a simple example: the accuracy of ENet is 58.3, the parameters are 0.4, the ratio of accuracy to parameters is 145.75, the accuracy of DABNet is 70.1, and the parameters are 0.76, then the accuracy and parameters ratio is 92.27. We know that DABNet is recognized as a network with much better performance than ENet. However, the ratio of accuracy to parameters is higher than DABNet, which shows that the relationship between accuracy and parameters is not *y* = *a* × *x*. There is an offset b between them. The relationship is *y* = *a* × (*x* + *b*). We have sorted out the formula:


(4) a=y/(x + b)

Among them, *y* represents the segmentation accuracy (mIoU), *x* represents the parameters (M), *b*represents the offset, and *a* represents the increment rate (IR). We bring the accuracy of PIDNet-S, 78.8 mIoU and parameter 7.6 M, and the accuracy of 70.1 mIoU and parameter 0.76 M of DABNet, which are recognized as the best lightweight real-time semantic segmentation at this stage, into the formula, and get *b* = 53.4. In this article, we take *b* = 53.4. The calculation formula of the IR is:


(5) a=y/(x+53.4)

### 4.3 Ablation study

#### 4.3.1 Ablation study for MS-DAB module

##### 4.3.1.1 Ablation for residual module

The encoder part of LMDCNet we designed uses the MS-DAB module. To prove the effectiveness of our designed encoder module, we compare the same type’s residual modules. We replace the MS-DAB module with a non-bottleneck-1D module, a ShuffleNet module, and a DAB module and test them on the Cityscapes dataset. As seen in Table 2, the LMDCNet network with ShuffleNet’s coding module has the lowest number of parameters and the fastest inference speed but the lowest segmentation accuracy. The combined consideration needs to be more competent for the actual segmentation task. On the other hand, the semantic segmentation network using MS-DAB has 0.15 M higher parameters than that using DAB, and the segmentation accuracy is improved by 4.1%, which is a more reasonable performance. The MS-DAB module we designed perfectly balances segmentation accuracy and parameters.

**TABLE 2:** Ablation study results of depth-asymmetric bottleneck module with multi-dimensional dynamic convolution and shuffling operations (MS-DAB module).

Type	Model	mIoU (%)	FPS	Params (M)
Baseline	LMDCNet	73.8	71.2	1.05
Ablation for residual module	LMDCNet-Non-bottleneck-1D	68.4	74.3	1.90
	LMDCNet-DAB	69.7	84.1	0.90
	LMDCNet-ShuffleNet	66.8	97.3	0.56
Ablation for dilation rates	4,4,4,4,4,4,4	72.3	70.8	1.05
	2,2,5,5,9,9,17	72.9	70.8	1.05
	2,2,4,4,8,8,16	73.2	70.9	1.05
Ablation for actiation function	Relu	73.4	70.9	1.05
Ablation for convolution	1D	70.2	73.2	1.01
	Cond-Conv	71.6	71.0	1.08
	Dy-Conv	72.8	71.5	1.04

##### 4.3.1.2 Ablation for dynamic convolution

We gradually replaced the multi-dimension dynamic convolution in MS-DAB with the factorial and dynamic convolution to confirm that the MDy-Conv we proposed has better experimental results than other convolutions displayed in Figure 3. Table 2 shows that the convolution with the fewest parameters and the fastest inference speed when utilizing the factorial convolution also has the least accurate segmentation. Even though there were 0.01 M more parameters with the MDy-Conv than with the Dynamic convolution module, segmentation accuracy increased by 1.0%, demonstrating the excellent efficiency of our MDy-Conv.

##### 4.3.1.3 Ablation for dilation rates

The size of the perceptual field of the network affects the segmentation accuracy of the network, and the lightweight real-time network uses dilated convolution to improve the receptive field of the network. A reasonable dilation rate can improve the segmentation accuracy of the network while avoiding grid problems. In order to verify whether the criterion for the dilation rates we designed is correct, we designed four groups of dilation rates for tuning. The dilation rates of the first two stages of our coding part are set to 1, and the third part is set to (4,4,4,4,4,4,4), (2,2,5,5,9,9,17), (2,2,4,4,8,8,16), and (1,2,5,2,5,9,17), respectively. The results from Table 2 show that the segmentation accuracy is the lowest when the dilation rate is set to (2,2,4,4,8,8,16), and the segmentation accuracy is the largest when it is set to (1,2,5,2,5,9,17). Experiments show that the design requirements for the dilation rate of our network should follow the HDC (hybrid dilated convolution) design principle. We design the final dilation rate of the network as: (1, 2, 5, 2, 5, 9, 17).

##### 4.3.1.4 Ablation for activation function

The introduction of nonlinear functions in the network can improve the network performance. The commonly used nonlinear functions in semantic segmentation are Relu and PRelu. We use PRelu in the baseline network and Relu in the comparison network. From the experimental results in Table 2, it is concluded that PRelu is more suitable for the LMDCNet network.

#### 4.3.2 Ablation study for SC-FP module

##### 4.3.2.1 Ablation for decoder module

The SC-FP module is the main component of the decoder in our LMDCNet, which is an integration of encoded features to refine the segmentation categories. However, most real-time semantic segmentation deletes the decoder to pursue inference speed. We replaced the SC-FP module in LMDCNet with 1 × 1 point convolution to justify the design of the SC-FP decoder module. Table 3 shows that 5.2 FPS improves the inference speed of the LMDCNet network with 1 × 1 convolution with 0.01 M parameter reduction, but the segmentation accuracy is decreased by 1.8%. In summary, our design of SC-FP is reasonable.

**TABLE 3:** Ablation study results of feature pyramid with spatial and channel attention (SC-FP module).

Type	Model	mIoU (%)	FPS	Params (M)
Baseline	LMDCNet	73.8	71.2	1.05
Ablation for decoder depth	LMDCNet-1 × 1	72.0	76.4	1.04
Ablation for attention	LMDCNet-CA	73.3	72.9	1.05
	LMDCNet-SA	73.1	73.2	1.05
Ablation for FP kernel size	LMDCNet-K333	73.1	73.2	1.05
	LMDCNet-K235	73.4	72.7	1.05
	LMDCNet-K135	72.8	72.9	1.05

##### 4.3.2.2 Ablation for channel attention

In designing SC-FP, we utilized the channel attention technique. To illustrate the appropriateness of choosing the channel attention branch in our SC-FP module, we removed the channel attention branch. Table 3 shows that the segmentation accuracy obtained by the decoding module without channel attention is 0.5% lower than that obtained using the SC-FP module. This experiment shows that the channel attention branch we designed can improve the segmentation accuracy of the network.

##### 4.3.2.3 Ablation for spatial attention

We introduce the spatial attention branch in SC-FP, and spatial attention focuses more on the spatial information of segmented targets to improve segmentation accuracy. To demonstrate the role of spatial branching in the decoder, we removed the spatial attention branch for comparison. Table 3 shows that the segmentation accuracy obtained by the decoder module without spatial attention is 0.7% lower than that obtained using the SC-FP module. This test shows that our spatial attention branch can improve the network’s ability.

##### 4.3.2.4 Ablation for kernel size

We employ convolutions with kernel sizes of 3 × 3, 5 × 5, and 7 × 7 to obtain various context information scales in the SC-FP module’s feature pyramid structure. We use a 3 × 3 kernel (K333) to replace each of the SC-FP module’s three convolutions to show how effective this method is. Table 3 displays the experimental results. Additionally, we set up two convolution combinations with smaller kernel sizes: 1 × 1, 3 × 3, 5 × 5 (i.e., K135) and 2 × 2, 3 × 3, 5 × 5 (i.e., K235). Table 3 demonstrates that our SC-FP module performs well when 3 × 3, 5 × 5, and 7 × 7 convolutions are used to construct a feature pyramid structure.

### 4.4 Evaluation results on cityscapes

The parameters of our designed LMDCNet are 1.05 M, the inference speed on a 1080Ti is 72.1FPS, the segmentation accuracy is 73.8 mIoU, and the increase rate is 1.36. The increment rate represents the balance between the accuracy and parameters of lightweight real-time semantic segmentation, and the larger the increment rate, the better the balance. As can be seen from Table 4, our increase rate is the highest among lightweight real-time semantic segmentation at this stage. The current state-of-the-art lightweight semantic segmentation network PIDNet-S has a growth rate of 1.29, which is smaller than that of the semantic segmentation network we designed. It can be seen that our designed network outperforms PIDNet-S in the balance between accuracy and parameters. The speed of our designed network is 46.1FPS faster than that of SFNet tested on the same 1080Ti platform. The number of parameters is only 1/12 of SFNet. Among the semantic segmentation network with an input resolution of 512 × 1024, our accuracy is the highest, 1.9 mIoU higher than LEANet.

**TABLE 4:** Evaluation results of our lightweight multi-dimensional dynamic convolutional network (LMDCNet) and other state-of-the-art real-time semantic segmentation models on the Cityscapes test set.

Model	Input size	Pretrain	GPU	mIoU (%)	FPS	Params (M)	IR
SegNet	640 × 360	ImageNet	TitanX	57	16.7	29.5	0.69
ENet	640 × 360	No	TitanX	58.3	135.4	0.4	1.08
ICNet	1024 × 2048	ImageNet	TitanX	69.5	30.3	26.5	0.87
ERFNet	512 × 1024	No	TitanX	68	41.7	2.1	1.23
ESPNet	512 × 1024	No	TitanX	60.3	112	2.1	1.09
BiSeNet	768 × 1536	ImageNet	TitanX	68.4	72.3	5.8	1.16
Fast-SCNN	1024 × 2408	ImageNet	TitanX	68	123.5	1.11	1.25
ESPNetV2	512 × 1024	No	TitanX	66.2	67	1.25	1.21
DFANet	512 × 1024	ImageNet	TitanX	70.3	160	7.8	1.15
LEDNet	512 × 1024	No	1080Ti	69.2	71	0.94	1.27
ESNet	512 × 1024	No	1080Ti	69.1	63	1.66	1.25
DABNet	512 × 1024	No	1080Ti	70.1	104	0.76	1.29
FDDWNet	512 × 1024	No	2080Ti	71.5	60	0.8	1.32
BCPNet	512 × 1024	No	TitanX	68.4	250.4	0.61	1.27
DDPNet	768 × 1536	No	1080Ti	74.0	85.4	2.52	1.32
LEANet	512 × 1024	No	1080Ti	71.9	77.3	0.74	1.35
SFNet	1024 × 2048	No	1080Ti	78.9	26	12.87	1.19
PIDNet-S	1024 × 2048	No	3090	78.8	93.2	7.6	1.29
LMDCNet	512 × 1024	No	1080Ti	73.8	72.1	1.05	1.36

**FIGURE 6:**
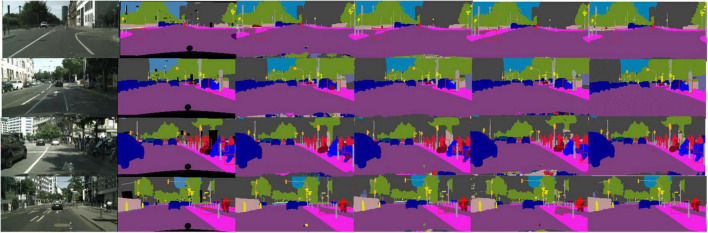
Some visual comparisons on the Cityscapes validation set. From left to right are input images, ground truth, predicted results from DABNet, FDDWNet, LEANet, and our LMDCNet.

We show the results for each class IoU (%) and class mIoU (%) on the Cityscapes test set in Table 5. Overall, especially in 5 categories, our LMDCNet achieves higher segmentation accuracy, demonstrating the effectiveness of our LMDCNet. Figure 6 shows a visual comparison of the Cityscapes validation set. We can classify different objects more accurately using LMDCNet and produce more consistent visual outputs across all categories. LMDCNet outperforms ERFNet, DABNet, and FDDWNet in the segmentation of vehicles, riders, and traffic signs.

**TABLE 5:** Evaluation results of each class Intersection over Union (IoU) (%) and class mIoU (%) on the Cityscapes test set.

Model	Ro	Si	Bui	Wa	Fe	Po	Tl	Ts	Ve	Te	Sk	Pe	Ri	Ca	Tru	Bus	Tr	Mo	Bi	Cl
SegNet	96.4	73.2	84.0	28.4	29.0	35.7	39.8	45.1	87.0	63.8	91.8	62.8	42.8	89.3	38.1	43.1	44.1	35.8	51.9	57.0
ENet	96.3	74.2	75.0	32.2	33.2	43.4	34.1	44.0	88.6	61.4	90.6	65.5	38.4	90.6	36.9	50.5	48.1	38.8	55.4	58.3
ICNet	97.1	79.2	89.7	43.2	48.9	61.5	60.4	63.4	91.5	68.3	93.5	74.6	56.1	92.6	51.3	72.7	51.3	53.6	70.5	69.5
ERFNet	97.7	81.0	89.8	42.5	48.0	56.3	59.8	65.3	91.4	68.2	94.2	76.8	57.1	92.8	50.8	60.1	51.8	47.3	61.7	68.0
Fast-SCNN	97.9	81.6	89.7	46.4	48.6	48.3	53.0	60.5	90.7	67.2	94.3	74.0	54.6	93.0	57.4	65.5	58.2	50.0	61.2	68.0
ESPNet	97.0	77.5	76.2	35.0	36.1	45.0	35.6	46.3	90.8	63.2	92.6	67.0	40.9	92.3	38.1	52.5	50.1	41.8	47.2	60.3
ESPNetV2	97.3	78.6	88.8	43.5	42.1	49.3	52.6	60.0	90.5	66.8	93.3	72.9	53.1	91.8	53.0	65.9	53.2	44.2	59.9	66.2
LEDNet	97.1	78.3	90.4	46.5	48.1	60.9	60.4	71.1	91.2	60.0	93.2	74.3	51.8	92.3	61.0	72.4	51.0	43.3	70.2	69.2
ESNet	97.1	78.5	90.4	46.5	48.1	60.1	60.4	70.9	91.1	59.9	93.2	74.3	51.8	92.3	61.0	72.3	51.0	43.3	70.2	69.1
DABNet	97.9	82.0	90.6	45.5	50.1	59.3	63.5	67.7	91.8	70.1	92.8	78.1	57.8	93.7	52.8	63.7	56.0	51.3	66.8	70.1
FDDWNet	98.0	82.4	91.1	52.5	51.2	59.9	64.4	68.9	92.5	70.3	94.4	80.8	59.8	94.0	56.5	68.9	48.6	55.7	67.7	71.5
LEANet	98.1	82.7	91.0	51.0	53.2	58.8	65.9	70.3	92.5	70.5	94.3	81.6	59.9	94.1	52.3	68.2	57.2	55.5	69.8	71.9
LMDCNet	98.2	82.7	91.2	51.4	53.1	59.3	65.8	70.5	92.6	70.2	94.2	81.5	59.8	94.2	52.9	68.1	57.7	55.7	69.7	73.8

### 4.5 Evaluation results on CamVid

Tables 6, 7 show the contrast between LMDCNet and other real-time semantic segmentation models for the CamVid dataset. Our LMDCNet produced effective segmentation results on the CamVid dataset. Without any prior training, our LMDCNet has a segmentation accuracy of 69.6 mIoU. Our LMDCNet can process 360 × 480 images at 92.4 FPS using a 1080Ti GPU for inference speed. In contrast to most real-time semantic segmentation models, LMDCNet has several clear advantages: fewer parameters, excellent segmentation accuracy, and quick inference speed. Our LMDCNet’s performance on the CamVid dataset is the best, illustrating its superior adaptability and effectiveness.

**TABLE 6:** Evaluation results of our lightweight multi-dimensional dynamic convolutional network (LMDCNet) and other state-of-the-art real-time semantic segmentation models on the CamVid test set.

Model	Input size	Pretrain	GPU	mIoU (%)	FPS	Params (M)
SegNet	360 × 480	ImageNet	TitanX	55.6	–	29.5
ENet	360 × 480	No	TitanX	51.3	–	0.4
ICNet	720 × 960	ImageNet	TitanX	67.1	27.8	26.5
CGNet	360 × 480	No	2xV100	65.6	–	0.5
BiSeNet	720 × 960	ImageNet	TitanX	65.6	175	5.8
BiSeNetV2	720 × 960	ImageNet	TitanX	68.7	124.5	49.0
DFANet	720 × 960	ImageNet	TitanX	64.7	120	7.8
DABNet	360 × 480	No	1080Ti	66.2	124.4	0.76
LRNNet	360 × 480	No	1080Ti	67.6	83	0.67
DDPNet	360 × 480	No	1080Ti	67.3	–	1.1
LEANet	360 × 480	No	1080Ti	67.5	98.6	0.74
LMDCNet	360 × 480	No	1080Ti	69.6	92.4	1.04

**TABLE 7:** Evaluation results of each class Intersection over Union (IoU) (%) and class mIoU (%) on the CamVid test set.

Mode	Bu	Tr	Sk	Ca	Si	Ro	Pe	Fe	Po	Si	Bi	Cl
SegNet	88.8	87.3	92.4	82.1	20.5	97.2	57.1	49.3	27.5	84.4	30.7	55.6
ENet	74.7	77.8	95.1	82.4	51.0	95.1	67.2	51.7	35.4	86.7	34.1	51.3
BiSeNet	82.2	74.4	91.9	80.8	42.8	93.3	53.8	49.7	25.4	77.3	50.0	65.6
BiSeNetV2	83.0	75.8	92.0	83.7	46.5	94.6	58.8	53.6	31.9	81.4	54.0	68.7
DABNet	80.8	73.3	91.0	81.0	40.0	94.8	59.5	56.6	29.8	80.3	41.7	66.2
LEANet	82.0	75.0	91.2	83.2	44.2	94.9	63.2	55.7	30.2	81.1	41.9	67.5
LMDCNet	82.7	76.3	91.7	83.5	46.6	94.5	59.0	53.9	32.4	81.7	53.9	69.6

## 5 Conclusion

We present a lightweight multi-dimension dynamic convolutional network (LMDCNet) with an ideal trade-off between model size, segmentation accuracy, and inference speed for real-time semantic segmentation. A multi-dimension dynamic convolution is what we suggest (MDy-Conv). In order to improve convolution presentation and maintain remarkable accuracy, it uses multi-convolutional kernel fusion. Our encoder is a depth-wise asymmetric bottleneck module with multi-dimension dynamic convolution and shuffling operations (MS-DAB module). This module can collect local and contextual information with fewer parameters and less computation. We propose a feature pyramid module (SC-FP module) based on spatial and channel attention for decoding. With minimal computational overhead, this module aggregates context data and generates pixel-level spatial and channel attention to aid in feature selection. According to experiments, our LMDCNet performs exceptionally well with the Cityscapes and CamVid datasets, making it the best option for various road scene interpretation applications.

## Data availability statement

Publicly available datasets were analyzed in this study. This data can be found here: https://www.cityscapes-dataset.com/; http://mi.eng.cam.ac.uk/research/projects/VideoRec/CamVid/.

## Author contributions

CZ, FX, CW, and CX were performed material preparation, data collection, and analysis. CZ wrote the first draft of the manuscript. All authors contributed to the study conception and design, commented on previous versions of the manuscript, read, and approved the final manuscript.
